# EC-QCL mid-IR transmission spectroscopy for monitoring dynamic changes of protein secondary structure in aqueous solution on the example of β-aggregation in alcohol-denaturated α-chymotrypsin

**DOI:** 10.1007/s00216-016-9464-5

**Published:** 2016-03-23

**Authors:** Mirta R. Alcaráz, Andreas Schwaighofer, Héctor Goicoechea, Bernhard Lendl

**Affiliations:** Institute of Chemical Technologies and Analytics, Vienna University of Technology, Getreidemarkt 9/164-UPA, 1060 Vienna, Austria; Laboratorio de Desarrollo Analítico y Quimiometría, FBCB, Universidad Nacional del Litoral-CONICET, Ciudad Universitaria, 3000 Santa Fe, Argentina

**Keywords:** Quantum cascade laser, Infrared spectroscopy, Multivariate curve resolution-alternating least squares, Protein secondary structure, Aggregation, 2,2,2-Trifluoroethanol

## Abstract

In this work, a novel EC-QCL-based setup for mid-IR transmission measurements in the amide I region is introduced for monitoring dynamic changes in secondary structure of proteins. For this purpose, α-chymotrypsin (aCT) acts as a model protein, which gradually forms intermolecular β-sheet aggregates after adopting a non-native α-helical structure induced by exposure to 50 % TFE. In order to showcase the versatility of the presented setup, the effects of varying pH values and protein concentration on the rate of β-aggregation were studied. The influence of the pH value on the initial reaction rate was studied in the range of pH 5.8–8.2. Results indicate an increased aggregation rate at elevated pH values. Furthermore, the widely accessible concentration range of the laser-based IR transmission setup was utilized to investigate β-aggregation across a concentration range of 5–60 mg mL^−1^. For concentrations lower than 20 mg mL^−1^, the aggregation rate appears to be independent of concentration. At higher values, the reaction rate increases linearly with protein concentration. Extended MCR-ALS was employed to obtain pure spectral and concentration profiles of the temporal transition between α-helices and intermolecular β-sheets. Comparison of the global solutions obtained by the modelled data with results acquired by the laser-based IR transmission setup at different conditions shows excellent agreement. This demonstrates the potential and versatility of the EC-QCL-based IR transmission setup to monitor dynamic changes of protein secondary structure in aqueous solution at varying conditions and across a wide concentration range.

**Electronic supplementary material:**

The online version of this article (doi:10.1007/s00216-016-9464-5) contains supplementary material, which is available to authorized users.

**Graphical abstract:**
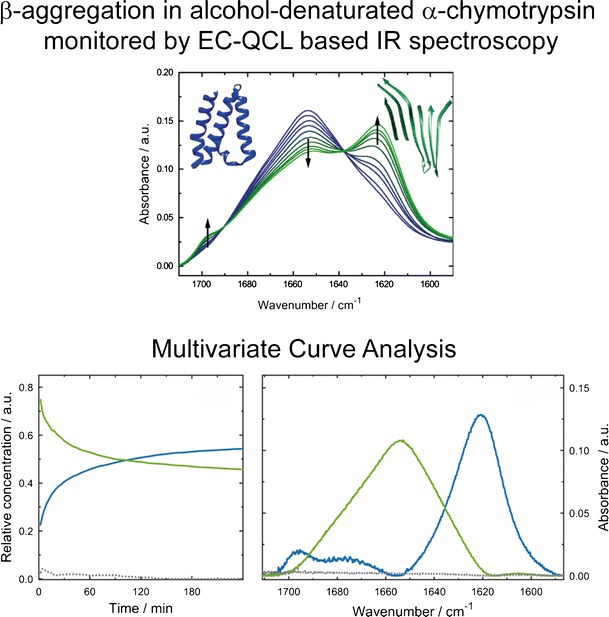
EC-QCL IR spectroscopy for monitoring protein conformation change

## Introduction

Infrared spectroscopy is a powerful and established analytical method to study the structure of proteins [1]. The most prominent absorption feature of proteins in the mid-IR region is the amide I band (1600–1700 cm^−1^) which is induced by vibrations of the peptide group. It is commonly used for analysis of protein secondary structure [2], because differing patterns of hydrogen bonding, dipole-dipole interactions and geometric orientations in the α-helices, β-sheets, turns and random coil structures induce different frequencies of the C=O vibrations that can be correlated with the respective secondary structural folding [3]. For adsorption studies or investigation of protein thin films, the attenuated total reflectance (ATR) technique is most commonly employed, while transmission measurements are routinely used for spectra acquisition of proteins in solution.

An experimental limitation to investigations of protein secondary structure in aqueous solutions with state-of-the-art Fourier transform infrared (FT-IR) spectrometers is constituted by the low feasible path lengths of transmission cells. This constraint originates from the combination of two factors: the high molar absorption coefficient of the HOH bending band of water near 1645 cm^−1^ that overlaps with the protein amide I band and the low emission power provided by the thermal light sources (globars) that are used in FT-IR spectrometers. As a consequence, path lengths most commonly used for IR transmission measurements of proteins in aqueous solutions are in the range of 7 μm to avoid total IR absorption in the region of the HOH bending band. This limitation comes along with laborious cell and sample handling as well as the need for high protein concentration (>10 mg mL^−1^) [2].

With the introduction of quantum cascade lasers (QCL), a significant step was made towards resolving the restrictions due to low-power light sources in mid-IR spectroscopy [4]. These new light sources provide emission powers that are several orders of magnitude higher than thermal IR sources and even offer brilliance values higher than reached by synchrotrons [5]. QCLs are unipolar lasers based on inter sub-band transitions of electrons within the semiconductors conduction band. Contrary to conventional semiconductor lasers, in QCLs, the emission wavelength range is decoupled from the band gap of the available semiconductor materials and depends primarily on the thicknesses of the semiconductor layers in the nanometer range. Only a few years ago, a new generation of QCLs, external-cavity QCLs (EC-QCLs), became commercially available, which are operated at room temperature and combine high emission power with a spectral tuning range of a few hundred wavenumbers. This large accessible spectral range permits the analysis of liquid samples, where absorption bands generally are broad and often show overlapping spectral features. The high emission power enables increased optical path lengths for transmission measurements even in the presence of strong absorbers such as water and promise benefits in terms of robustness and sensitivity. Thus, this type of QCL has been increasingly used for liquid phase samples and has been successfully applied for analysis of complex mixtures of analytes in aqueous solution in online process monitoring [6] and for medical applications [7–9]. Most recently, EC-QCL-based IR transmission measurements have been accomplished for the analysis of protein secondary structure [10]. It has been shown that the protein spectra recorded with the laser-based setup show excellent comparability with spectra acquired by FT-IR spectroscopy. Identification of spectral features of different secondary structures at protein concentrations as low as 2.5 mg mL^−1^ has been achieved through rigorous application of an advanced data processing protocol which was established to overcome noise issues resulting from mechanical imperfections of the tuning mechanism and the fine structure of the EC-QCL emission curve.

IR spectroscopy is frequently used for studying dynamic changes of protein secondary structure. Alterations of secondary structure can be induced by changing external conditions such as pH, temperature, pressure, co-solvents, surfactants, or chaotropic agents, which is often accompanied by protein denaturation. The most prominent change of secondary structure is the transition of α-helix to β-sheet resulting from protein aggregation. While various types of denaturation are accompanied by disruption of α-helices and formation of β-sheets, turns and polyproline type II helices, exposure to alcohol induces and stabilizes α-helical domains [11]. The effect of alcohols, particularly those substituted with fluorine, on proteins has been extensively studied during the last years; however, the physical mechanisms by which it affects protein conformation are still unclear. In the case of 2,2,2-trifluoroethanol (TFE), its low dielectric constant (one third of water for pure solvent) is believed to weaken solvophobic interactions that stabilize the native structure of proteins, and simultaneously strengthen electrostatic interactions such as intermolecular hydrogen bonds, thereby stabilizing local secondary structures, particularly the α-helix [12]. In addition, it was suggested that fluorine-substituted alcohols form large micelle-like clusters of alcohol molecules, resulting in a high local alcohol concentration. The strong electron-withdrawing effect of fluorine atoms makes TFE a better hydrogen bond donor, but a poorer acceptor, compared to water. Upon binding to these hydrophobic clusters, proteins and peptides undergo conformational transitions [13]. Overall, the detailed effects of TFE on protein conformation have found to be diverse and strongly dependent on the concentration ranges of the protein and the co-solvent [14].

α-Chymotrypsin (aCT) is a predominantly β-sheet protein folded in two antiparallel β-barrel domains with a molecular weight of 25 kDa and an isoelectric point (pI) at 8.4 [15, 16]. The predisposition of aCT towards amyloid fibril aggregation at intermediate TFE concentrations (15–35 %) and low protein concentrations (0.025–0.5 mg mL^−1^, 1–20 μmol L^−1^) has been investigated by turbidimetric, dynamic light scattering, thermodynamic, intrinsic fluorescence and quenching studies [17–19]. It has been shown that the interaction greatly depends on solution conditions such as pH [17], TFE concentration [17, 18], protein concentration [17] and temperature [17, 19]. Amyloid fibril formation is promoted at intermediate TFE concentrations, but at higher concentrations it is suppressed due to pronounced stabilization of non-native α-helical structures [20]. For high protein concentrations (20–30 mg mL^−1^, 800–1200 μmol L^−1^), it has been shown that exposure of aCT to high TFE concentrations (50 %) leads to instantaneous formation of non-native α-helical structures by employing FT-IR and CD spectroscopy. This fast transition is succeeded by gradual formation of intermolecular β-sheet aggregates. At these conditions, proteins consisting of native α-helical secondary structure do not show β-sheet aggregation after contact with TFE [21].

Multivariate curve resolution-alternating least square (MCR-ALS) is a widespread iterative soft-modelling method introduced by Tauler in 1995 [22]. This technique allows obtaining information about multicomponent systems by discriminating individual contributions of underlying constituents [23]. Nowadays, MCR-ALS has demonstrated to be a powerful chemometric tool to overcome different chemical problems in several analytical fields. Due to its flexibility and robustness, this method has been successfully used in combination with various analytical techniques, such as chromatography [24], electrophoresis [25], flow analysis [26] and infrared spectroscopy [27–29]. For analysis of complex data matrices, extended MCR-ALS is applied in order to significantly decrease the ambiguity of the resolution and overcome problems associated with rank-deficiency. In this study, extended MCR-ALS is applied allowing to analyse multiple experiments simultaneously and a global solution is achieved [30, 31].

The aim of this work is to establish the recently introduced EC-QCL mid-IR transmission setup as a tool for monitoring dynamic changes of protein secondary structure. To this end, aCT was selected as a model protein which shows a gradual transition from α-helix to intermolecular β-sheet after exposure to TFE. In order to showcase the potential and versatility of the presented setup, the effects of varying pH values and protein concentration on the rate of β-aggregation were investigated. The wide accessible concentration range of the laser-based IR transmission setup was employed to study β-aggregation across a concentration range of 5–60 mg mL^−1^ (0.2–2.4 mmol L^−1^). Furthermore, the influence of the pH value on the initial reaction rate was studied in the range of pH 5.8–8.2. Extended MCR-ALS was used to obtain pure spectral and concentration profiles of the temporal transition between α-helices and intermolecular β-sheets.

## Materials and methods

### Reagents and samples

Sodium phosphate monobasic dihydrate p.a. (NaH_2_PO_4_•2H_2_O) was purchased from Fluka (Buchs, Switzerland), sodium phosphate dibasic dihydrate (Na_2_HPO_4_•2H_2_O) BioUltra, for molecular biology, sodium hydroxide solution 50 % in water (NaOH), hydrochloric acid 37 % (HCl) ACS reagent and 2,2,2-trifluoroethanol ReagentPlus ≥99 % (TFE), were obtained from Sigma-Aldrich (Steinheim, Germany). α-Chymotrypsin from bovine pancreas (≥85 %) (aCT) was obtained by Sigma-Aldrich (Steinheim, Germany) and used as purchased. Ultrapure water (18 MΩ cm) used for preparation of all solutions was obtained with a Milli-Q water purification system from Millipore (Bedford, USA).

All aCT protein solutions were prepared by dissolving an appropriate amount of lyophilized protein powder directly in 1.0 mL of a TFE/16.0 mmol L^−1^ phosphate buffer (50:50) mixture solution and placed immediately into the flow cell of the EC-QCL setup. The pH of the 16.0 mmol L^−1^ phosphate buffer solutions was adjusted with NaOH or HCl prior to the mixing with TFE.

A set of seven samples (protein concentration: 20 mg mL^−1^) was prepared at different pH values ranging between 5.8 and 8.2. Furthermore, a set of seven samples at pH 6.6 with final concentrations ranging between 5 and 60 mg mL^−1^ of aCT was prepared. pH measurements were carried out with a pH 330i (Wissenschaftlich-Technische Werkstätten GmbH, Weilheim, Germany) potentiometer equipped with a Sentix^®^ 61 (Wissenschaftlich-Technische Werkstätten GmbH, Weilheim, Germany) combined glass electrode and temperature probe.

### EC-QC laser setup

**Fig. 1:**
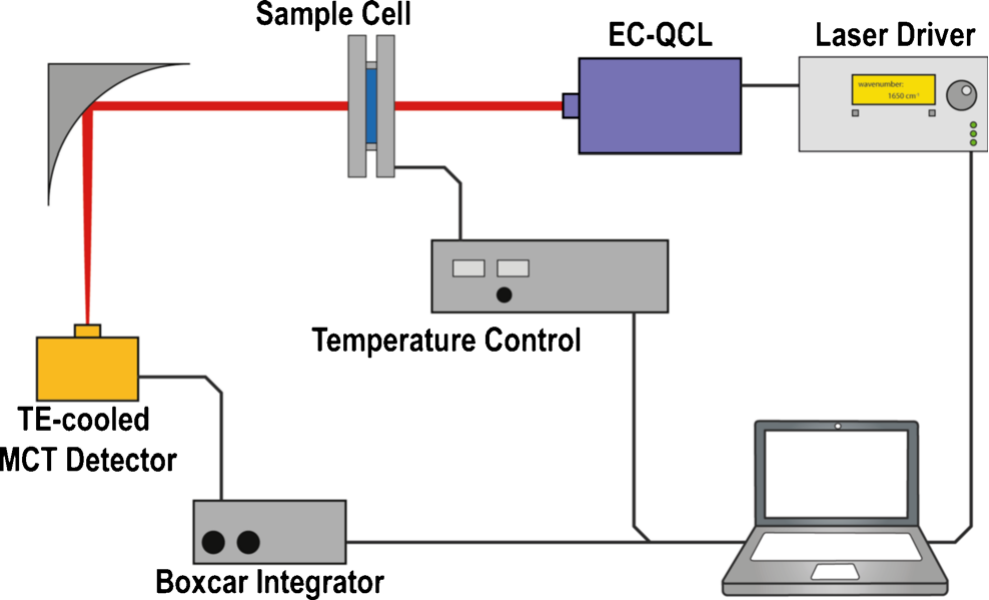
Schematic of the experimental QCL-based setup for mid-IR transmission measurements

IR measurements were performed on a custom-made EC-QCL setup equipped with a quantum cascade laser (Daylight Solutions Inc., San Diego, USA) with spectral tuning range between 1729.30 and 1565.06 cm^−1^, a temperature-controlled 38-μm path length flow cell and a thermoelectrically cooled MCT detector (Infrared Associates Inc., USA; MCT-7-TE3) as depicted in Fig. 1. The laser was thermoelectrically cooled and was operated in pulsed mode at a repetition rate of 100 kHz and a pulse width of 500 ns. The laser head temperature was set to 18 °C for all measurements. A gold plated off-axis parabolic mirror (focal length: 43 mm) was used to focus the MIR light on the detector operating at −60 °C with a 1 × 1 mm element size and a detectivity of D* = 4 × 10^9^ cm Hz^0.5^ W^−1^ at 9.2 μm. The measured signal was processed by a two-channel boxcar integrator and digitized by a NI DAQ 9239 24-bit ADC (National Instruments Corp., Austin, USA) at a sampling rate of 16 kHz. The whole setup was controlled by a LabView-based GUI 11.0 (National Instruments Corp., Austin, USA, 2011) with server–client program structure [32].

All measurements were conducted at 25 °C using a custom-made, temperature-controlled flow cell equipped with two MIR transparent CaF_2_ windows and 38 μm-thick spacer. To reduce the influence of water vapour, the setup was placed in a housing of polyethylene foil and constantly flushed with dry air. Each single beam spectrum consisting of 24,000 data points was recorded during the tuning time of 1.5 s. A total of 20 scans were recorded for background and sample single beam spectra (total measurement time for 20 scans: 100 s). The single beam spectra of the corresponding solvent were taken as reference and recorded under identical conditions as sample spectra. To minimize the spectral noise originating from the spectral mismatch of successive scans, the data processing routine based on Correlation Optimized Warping (COW) was employed to align spectra of repeated scans as well as of the background with the sample spectrum as described earlier [10]. At last, minor Fourier filtering was applied to remove residual noise in the final absorbance spectra.

OPUS 7.2 (Bruker Optik GmbH, Ettlingen, Germany, 2012) was used for spectral evaluation.

### MCR-ALS

MCR-ALS is a soft-modelling technique that focuses on bilinear decomposition of a data matrix **D** into two submatrices containing chemically meaningful information of contributions of the pure compounds involved in the system [33]. The decomposition is the result of the validity of Beer-Lambert’s Law and is achieved by iterative optimization according to the expression,

1 $$ \mathbf{D}\kern0.5em =\kern0.5em \mathbf{C}\times {\mathbf{S}}^{\mathrm{T}}\kern0.5em +\kern0.5em \mathbf{E} $$

where **C** contains the profiles referred to the abundance of the qualitative pure responses and **S**^T^ comprises the pure instrumental responses of the components in the system; **E** contains the residuals of the model [34].

One of the most intriguing characteristics of MCR-ALS resolution is its operation without prior information about the system under study. However, additional knowledge can be included in order to achieve chemically meaningful component profiles. Decomposition of **D** is obtained by iteratively optimizing the initial estimates of either **C** or **S** using the available knowledge about the system [30]. This information is introduced through the implementation of chemical or mathematical constraints, such as non-negativity, unimodality, normalization and closure, among others [35].

Here, protein β-aggregation was monitored at varying protein concentrations and pH values in the spectral range between 1710 and 1585 cm^−1^ during a time period of 240 min. The corresponding time-absorption spectra matrix for one measurement run consisted of 38 × 4600 data points for the temporal and spectral dimension, respectively. For MCR-ALS analysis, unfiltered spectra obtained after sample-background alignment were used to build the time-absorption spectra matrix. Baseline correction based on a multidimensional extension of the asymmetric least squares method proposed by Eilers [36] was applied prior to performing MCR-ALS.

All data sets belonging to the measurement series investigating either the pH- or concentration dependence of β-aggregation were merged in two individual augmented column-wise data matrices **D** by appending the time-absorption spectra matrices related to each experiment in the column direction. In this way, **D**_pH_ contained the pH-dependent experiments, and **D**_conc_ was built with the concentration-dependent experiments. Prior to MCR-ALS resolution, determination of the number of compounds in each data matrix **D** was carried out using singular value decomposition (SVD). Initial time-evolution estimations were obtained using a routine based on the simple iterative self-modelling approach (SIMPLISMA) methodology [37]. ALS optimization was carried out applying different constraints, i.e. non-negativity in both modes, unimodality in the temporal mode and normalization in spectral mode. After decomposition, the column profiles of matrix **C** and the row profiles of **S** were associated with the temporal evolution and pure spectra profiles of α-helix and β-sheet conformation of the protein, respectively.

Data processing and analysis as well as MCR-ALS were performed in MATLAB R2014b (MathWorks, Inc., Natick, MA, 2014). MCR-ALS algorithms are available online at http://www.mcrals.info/.

## Results and discussion

### Effect of TFE on the IR spectra of α-chymotrypsin

**Fig. 2:**
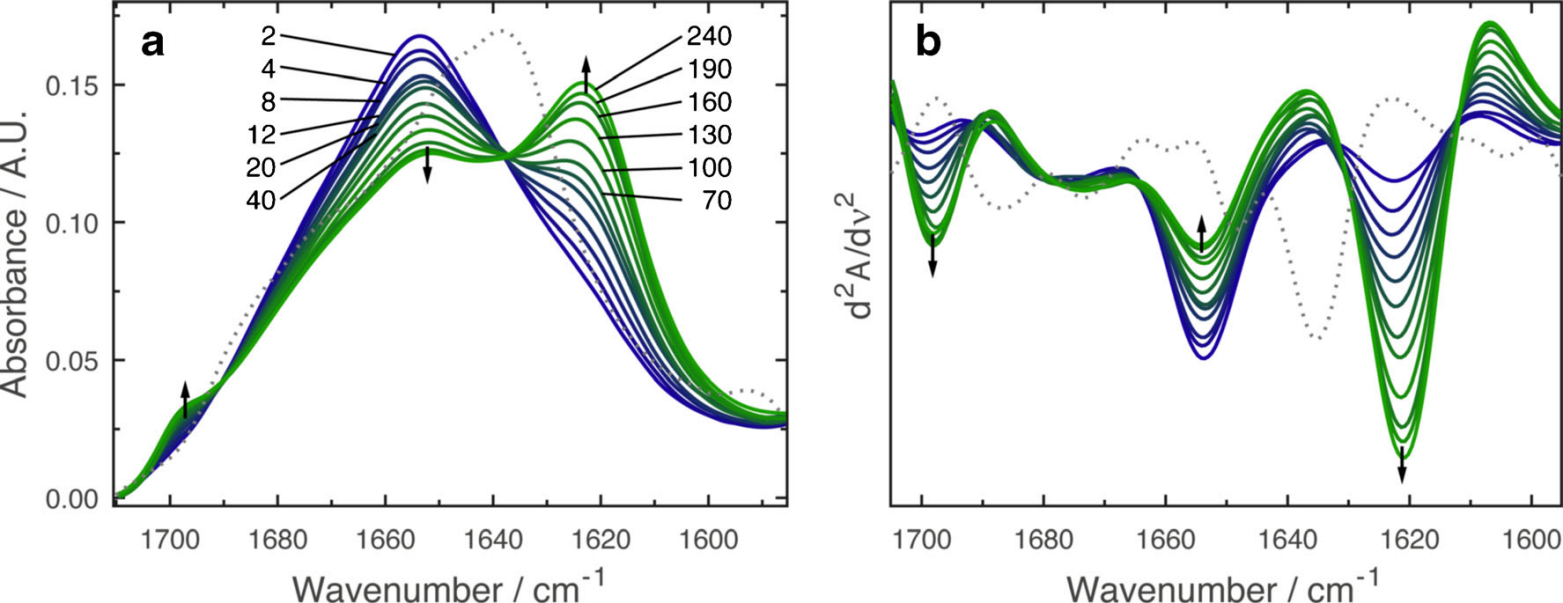
**a** Time-dependent IR absorbance and **b** second-derivative spectra of 20 mg mL^−1^ α-chymotrypsin in 50 % TFE/buffer solution, pH 7.8 at 25 °C (*solid lines*). The spectra were recorded at time periods between 2 and 240 min (times as indicated in the graph) after the protein was dissolved in TFE/buffer. *Blue solid lines* show the spectrum of aCT with TFE-induced α-helical structure. *Green solid lines* indicate the spectrum of the protein after gradual formation of intermolecular β-sheets. *Grey dashed lines* represent spectra of the native protein in aqueous buffer. *Black arrows* illustrate directions of absorbance changes as a function of time

Employing the EC-QCL setup, mid-IR transmission spectra were recorded of aCT in aqueous buffer solution and after exposure to 50 % TFE/buffer solution. In Fig. 2, the IR absorbance and second-derivative spectra are shown. The IR absorbance spectrum of native aCT shows a band maximum at 1638 cm^−1^ and a shoulder at 1680 cm^−1^, characteristic for the low- and high-frequency components in β-sheet secondary structure [38]. Upon exposure to 50 % TFE/buffer solution, the maximum of the amide I band changes to 1654 cm^−1^, typical for the formation of α-helical structures [38]. This effect is in accordance with earlier studies describing the generation of α-helical secondary structure in proteins after exposure to TFE [21, 39]. The TFE-induced transition from native β-sheet secondary structure to α-helix takes place in the time range of milliseconds [40] and is not directly observable with the employed setup.

It was observed that the TFE-induced α-helical secondary structure is not stable over time, as previously reported for proteins exhibiting native β-rich secondary structure [21]. Figure 2 shows the gradual change of the IR spectrum of aCT over a time period of 240 min. The intensity of the band at 1654 cm^−1^ decreases as bands at 1623 and 1697 cm^−1^ emerge. This arising spectral pattern is commonly attributed to intermolecular antiparallel β-sheets aggregates, frequently occurring in thermally denatured proteins [41, 42].

Investigations of proteins exhibiting predominantly α-helical secondary structure such as bovine serum albumin and myoglobin did not reveal spectral changes indicating intermolecular β-sheet formation over a comparable period of time (data not shown).

### pH dependence of β-aggregation

TFE-induced formation of intermolecular β-sheets of 20 mg mL^−1^ aCT in 50 % TFE/buffer solution was investigated in the range of pH 5.8–8.2. It is noteworthy that under the applied conditions (pH values and protein concentration range), no visible precipitation and gelation or increase of turbidity of the protein solution was observed within 24 h. That observation strongly suggests that the effects observed under the conditions of the present study differ from classical amyloid fibril aggregation as investigated in numerous studies [17–19]. This is in accordance with earlier reports which found that at high TFE concentrations, proteins appear in an aggregation deficient non-native state (also called TFE-state), that is not prone to form amyloid-like fibrils [17, 43].

**Fig. 3:**
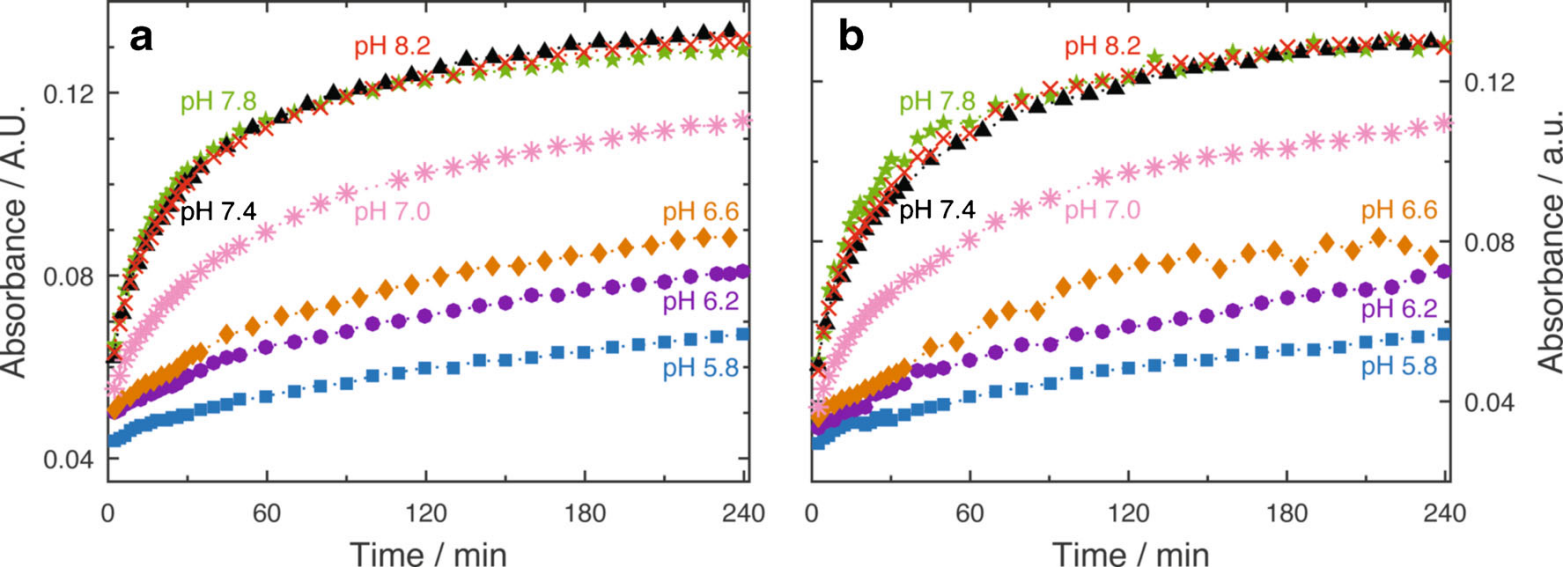
Temporal progression of the evolving β-sheet content of 20 mg mL^−1^ aCT in 50 % TFE/buffer solution at different pH values (pH 5.8–8.2) obtained by **a** evaluation of the IR absorbance spectra (absorbance at 1623 cm^−1^) and **b** MCR-ALS

**Fig. 4:**
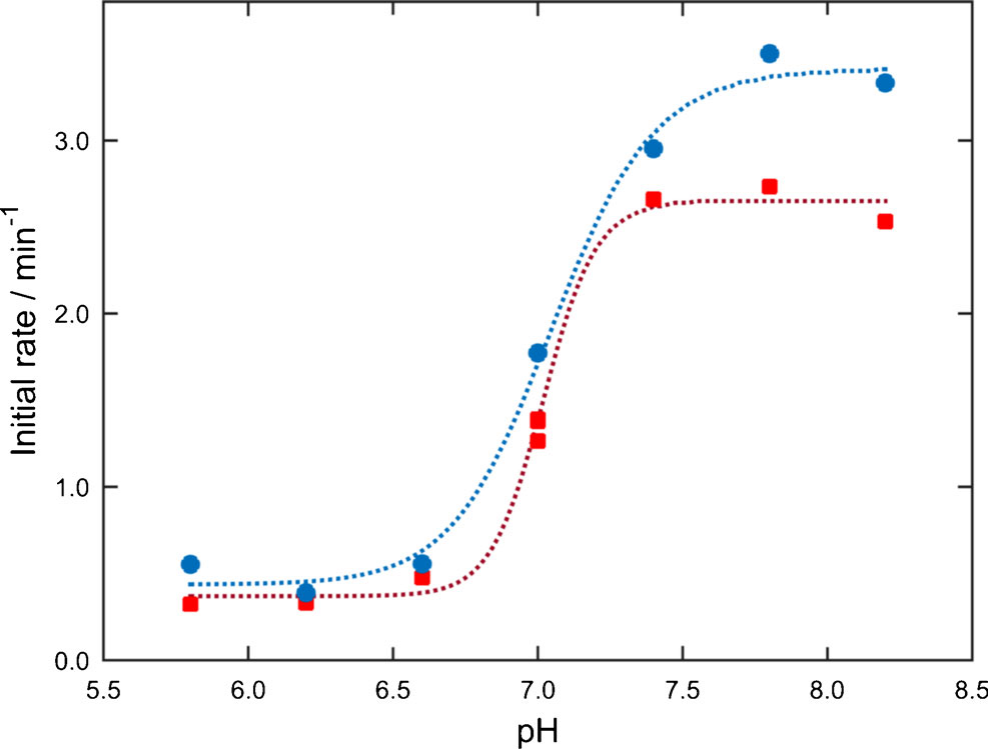
Effect of varying pH values on initial rate of β-sheet aggregation of 20 mg mL^−1^ aCT in 50 % TFE/buffer as analysed by (*red squares*) evaluation of the IR absorbance spectra and (*blue circles*) MCR-ALS

For analysis of the temporal progression of the evolving β-sheet content at different pH values, the intensity change relative to the baseline of the absorbance at 1623 cm^−1^ was evaluated (Fig. 3a). The temporal profiles clearly show the strong pH dependence of the β-sheet formation since the change of absorbance is higher at elevated pH values. For quantitative assessment of this behaviour, the initial rate was calculated as the slope of the absorbance changes throughout the first four measurement points (2–8 min) against time. Figure 4 shows the effect of varying pH values on the initial rate of β-sheet aggregation. The initial rate shows a sigmoidal progression with low values for mildly acidic pH and high values for basic pH, with the transition point at approximately pH 7.0. For higher pH values, the reaction appears to be finished within 4 h, whereas aggregation is not completed at lower pH values within the observed time period.

In general, proteins show lowest solubility at solution pH values near their isoelectric points. Under these conditions, proteins possess both positively and negatively charged groups, leading to an anisotropic charge distribution on the protein surface generating possible dipoles. Thus, protein-protein interactions could be highly attractive, rendering assembly processes such as aggregation energetically favourable [44, 45]. In aqueous buffer solution, no β-aggregation could be observed in the investigated pH range. Apparently, the electrostatic effect destabilizing the protein conformation is intensified by the addition of TFE. Here, this aspect is reflected by the increase of the initial rate of β-sheet formation at pH values close to the pI of the protein. In a previous study, a comparable change of aggregation behaviour near the pI was observed for amyloid fibril aggregation at intermediate TFE concentrations, i.e. with aCT appearing in the fibril aggregation prone state [17].

The reproducibility of the system was evaluated by monitoring the β-aggregation of a triplicate of 20 mg mL^−1^ aCT solution at pH 7.0. These particular experimental conditions were selected since at this pH and concentration level, the highest variation was expected, as shown in Fig. 4. The coefficient of variation of the initial rate at these conditions was determined to be 4.2 %, which certifies excellent reproducibility of the method for monitoring protein aggregation with an EC-QCL setup.

### β-aggregation monitored at different protein concentrations

**Fig. 5:**
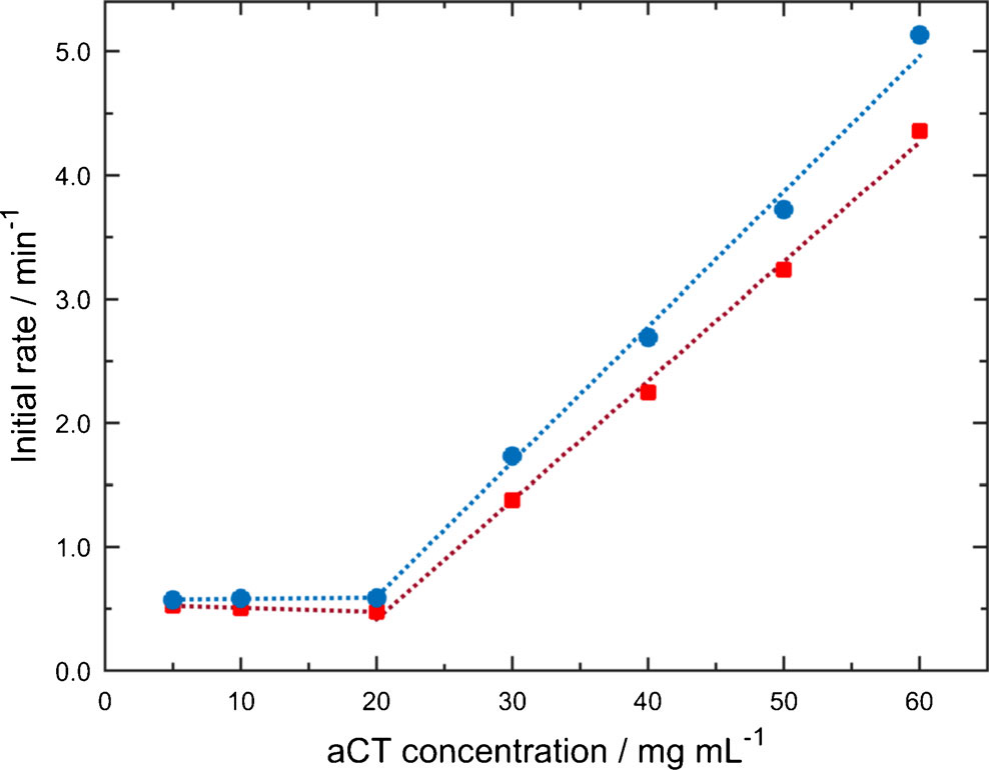
Initial rate of β-aggregation for different aCT concentrations in 50 % TFE/buffer at pH 6.6 as analysed by (*red squares*) evaluation of the IR absorbance spectra and (*blue circles*) MCR-ALS

To showcase the wide accessible concentration range of the laser-based IR transmission setup, β-sheet formation of aCT in 50 % TFE/buffer solution was monitored in a range between 5 and 60 mg mL^−1^ of protein at pH 6.6. Again, the initial rate was evaluated by calculating the slope of the first four measurement points (2–8 min) of the temporal progression against time. Figure 5 shows the effect of varying protein concentrations on the initial rate of β-aggregation. For protein concentrations between 5 and 20 mg mL^−1^, the values for the initial rate remain constant, whereas at higher values, the reaction rate increases linearly with protein concentration.

The results suggest that at low protein concentration, the aggregation rate is independent from concentration. The characteristic change of the slope at 20 mg mL^−1^ indicates the value of the critical concentration. This behaviour has been found for the aggregation characteristics of numerous proteins [45]. For protein concentrations higher than the critical concentration, the initial rate continually increases. This tendency agrees with results of an earlier study, where higher rates of change for β-aggregation with increasing protein concentrations have been found for aCT in the presence of 50 % TFE at pH 7.4 [21]. Generally, protein aggregation increases with higher protein concentration due to the higher probability of protein-protein association [45].

### Extended MCR-ALS analysis

Detailed chemometric analysis of the two QCL-IR transmission datasets of pH - and concentration-dependent β-aggregation was performed using extended MCR-ALS. For this study, MCR-ALS was chosen because it offers the possibility to reveal the pure spectral profiles for the compounds involved in the reaction as well as their temporal progressions without any prior knowledge of the system. Other chemometric methods such as parallel factor analysis (PARAFAC) also allow obtaining loadings with chemical interpretation about the system in terms of pure spectral profiles of the involved components. However, since in the current study the temporal evolution for different parameters is not identical, this algorithm is not applicable, due to a lack of trilinearity present in these datasets [34]. On the other hand, algorithms based on partial least square (PLS) are generally suitable for second-order calibration data analysis, but the results obtained as loadings and scores do not provide an approximation to the pure constituent profiles [46]. Thus, these algorithms are not applicable when spectral information is desired.

**Fig. 6:**
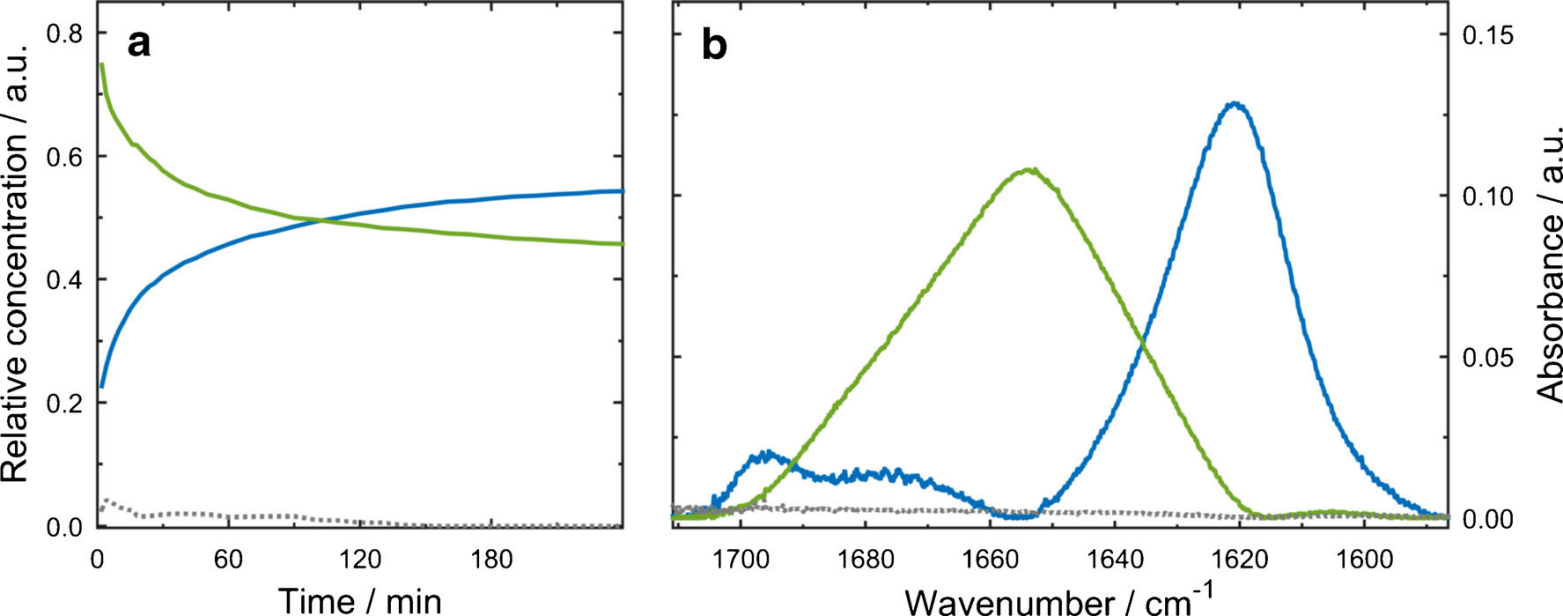
**a** Temporal and **b** spectral profiles retrieved by MCR-ALS for 20 mg mL^−1^ aCT in 50 % TFE/buffer solution at pH 8.2. *Solid green* and *solid blue* lines show individual contributions of α-helical and intermolecular β-sheet conformation, respectively. *Dashed grey* lines indicate the instrumental noise of the system

In the extended variant of MCR-ALS, multiple matrices are analysed simultaneously to reduce resolution ambiguities and rank-deficiency problems. Seven individual time-resolved IR measurements at different pH values were combined to an augmented data matrix to obtain the temporal evolution of β-aggregation (Fig. 6a) as well as spectral profiles (Fig. 6b) of the individual protein secondary structures elements involved in the process. The noise level of the spectral profiles appears low, in particular when considering that MCR-ALS modelling has been performed with spectra that have not been Fourier filtered. Values for lack of fit (LOF, 1.8 %) and percentage of explained variance (*R*^2^, 99.98 %) indicate good description of the experimental data by the MCR-ALS model. Here, three components were identified, two of them were involved in the aggregation process and one was referred to as instrumental noise. The obtained spectral profiles of pure spectra are consistent with band maxima and shapes of the associated protein secondary structure components. One component features a band maximum at 1654 cm^−1^ and is assigned to α-helical secondary structure [1]. Its decline of absorbance along the reaction time is in accordance with the decrease of α-helical content during β-aggregation (see Fig. 6a). The second component shows a strong band at 1621 cm^−1^ and a weaker band at 1695 cm^−1^, which is the typical spectral pattern associated with antiparallel intermolecular β-sheets [47]. A third component was attributed to instrumental noise. It does not show any characteristic features in the spectral profile or specific absorbance changes in the temporal profile.

Temporal profiles of the component assigned to intermolecular β-sheets at different pH values are plotted in Fig. 3b. Comparison of the modelled data with temporal progressions obtained by evaluation of IR spectra shows good agreement, which is, moreover, reflected in the similar trend regarding the pH dependence of initial rates (see Fig. 4).

The procedure of extended MCR-ALS was also performed for the measurement series studying the influence of initial protein concentration on β-aggregation. Values for LOF and *R*^2^ are 1.3 and 99.98 %, respectively, and prove excellent quality of the MCR-ALS analysis. Temporal and spectral profiles (see Electronic Supplementary Material Fig. S1) show three components similar to the results of MCR-ALS modelling of the pH-dependent study. The compatible outcome obtained by extended MCR-ALS analyses of two independent measurement series demonstrates the ruggedness of bilinear decomposition. Also here, the initial rates of β-aggregation were computed from modelled data and show a matching tendency with values obtained from evaluation of experimental IR spectra for increasing protein concentrations (see Fig. 5).

## Conclusion

Recently, an EC-QCL-based IR transmission setup has been introduced for application in secondary structure analysis of proteins in aqueous solution. The high optical power provided by the laser light source enables transmission measurements using higher path lengths (up to 38 μm) and protein concentrations as low as 2.5 mg mL^−1^. Using this laser-based setup, protein spectra recorded under static conditions showed excellent comparability with spectra acquired by FT-IR spectroscopy.

The aim of the present work is to employ the EC-QCL-based IR transmission setup to accomplish monitoring of dynamic changes in protein secondary structure. The gradual formation of intermolecular β-sheet aggregates after inducing non-native α-helical structures in aCT by exposure to TFE was monitored at varying pH values and protein concentrations. It has been shown that the initial reaction rate increases close to the pI of aCT, attributed to higher attractive electrostatic interactions under these conditions. The rate of β-aggregation has been found to increase linearly at protein concentration levels above the critical value. The observed results agree with the suggestion that TFE reduces hydrogen bonds formed between proteins and surrounding water molecules, thus inducing protein conformations that are compact and maximise intermolecular hydrogen bonding. The gradual formation of β-sheet aggregates from primarily generated α-helical structures induced by TFE is in accordance with earlier studies that found that α-helical structures represent the kinetically favoured state and intermolecular β-sheets constitute the thermodynamically preferred state of aCT under the investigated solvent conditions [21].

Extended MCR-ALS analysis of pH and concentration-dependent measurements show similar spectral profiles, demonstrating the high quality of the IR spectra obtained by the EC-QCL-based IR transmission setup at varying conditions and the general robustness of this chemometric technique. Concentration profiles obtained by the MCR-ALS model show good comparability with evaluation of IR spectra.

The present study demonstrates the high potential and great versatility of the laser-based IR transmission setup to monitor dynamic changes of protein secondary structure in aqueous solution. The use of a transmission flow cell with a path length approximately four times higher than usually employed for conventional FT-IR spectrometers facilitates experiments in flow-through configuration. Flow injection analysis (FIA) as well as sequential injection analysis (SIA) are established techniques that could be coupled to the presented laser-based IR transmission setup for denaturation studies of proteins and investigations of protein-ligand binding. For monitoring dynamic events on shorter time scale, stopped flow mixing is feasible. For time-resolved measurements, the wavenumber tuning rate of the EC-QCL constitutes the limiting factor. Typical rates for broadband tuning of commercially available lasers are in the range of 100 cm^−1^ s^−1^. Significantly higher time-resolution can be obtained by keeping the emission wavenumber of the laser constant throughout one experiment and incrementally recording the absorbance at individual wavenumbers along the reaction time during multiple measurement repetitions. Rearrangement of the recorded data obtains the set of time-resolved spectra. A prerequisite for this approach is that the observed sample reaction is repeatable as well as reproducible and following from that, the initiation of reagent mixing by an automated flow injection technique.

## Electronic supplementary material

Below is the link to the electronic supplementary material.

ESM 1 (PDF 168 kb)
