# Supplementary material for: EC-QCL mid-IR transmission spectroscopy for monitoring dynamic changes of protein secondary structure in aqueous solution on the example of β-aggregation in alcohol-denaturated α-chymotrypsin
Source: Anal Bioanal Chem. 2016 Mar 23;408:3933–41. doi: 10.1007/s00216-016-9464-5 (PMC4873525; doi:10.1007/s00216-016-9464-5)
Supplement: Supplementary file 1 — (PDF 168 kb) [file 216_2016_9464_MOESM1_ESM.pdf]

Analytical and Bioanalytical Chemistry

Electronic Supplementary Material

EC-QCL mid-IR transmission spectroscopy for monitoring dynamic changes of protein secondary structure in aqueous solution on the example of β-aggregation in alcohol-denaturated α-chymotrypsin

Mirta R. Alcaráz, Andreas Schwaighofer, Héctor Goicoechea, Bernhard Lendl

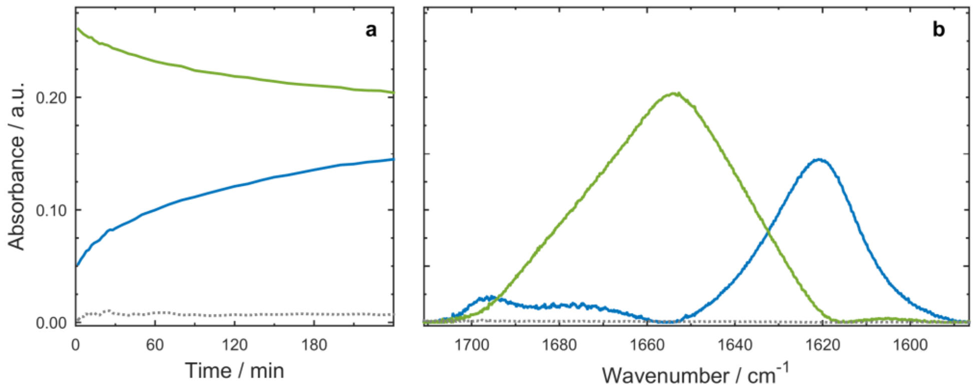

Fig. S1 (**a**) Temporal and (**b**) spectral profiles retrieved by extended MCR-ALS for 30 mg mL^{-1} aCT in 50 % TFE/buffer solution at pH 6.6. *Solid green* and *solid blue* lines show individual contributions of α-helical and intermolecular β-sheet conformation, respectively. *Dashed grey* lines indicate the instrumental noise of the system.
